# [^68^Ga]NODAGA-RGD – Metabolic stability, biodistribution, and dosimetry data from patients with hepatocellular carcinoma and liver cirrhosis

**DOI:** 10.1007/s00259-016-3396-3

**Published:** 2016-05-11

**Authors:** Roland Haubner, Armin Finkenstedt, Armin Stegmayr, Christine Rangger, Clemens Decristoforo, Heinz Zoller, Irene J. Virgolini

**Affiliations:** 1 Department of Nuclear Medicine, Medical University of Innsbruck, Anichstr. 35, 6020 Innsbruck, Austria; 2 Department of Internal Medicine II, Medical University of Innsbruck, Innsbruck, Austria; 3 FH Gesundheit/University of Applied Sciences Tyrol, Innsbruck, Austria

**Keywords:** [^68^Ga]NODAGA-RGD, PET, Metabolic stability, Dosimetry, Whole-body distribution, Hepatocellular carcinoma

## Abstract

**Purpose:**

This study was designed to determine safety, tolerability, and radiation burden of a [^68^Ga]NODAGA-RGD-PET for imaging integrin α_v_β_3_ expression in patients with hepatocellular carcinoma (HCC) and liver cirrhosis. Moreover, metabolic stability and biokinetic data were compiled.

**Methods:**

After injection of 154–184 MBq [^68^Ga]NODAGA-RGD three consecutive PET/CT scans were acquired starting 8.3 ± 2.1, 36.9 ± 2.8, and 75.1 ± 3.4 min after tracer injection. For metabolite analysis, blood and urine samples were analyzed by HPLC. For dosimetry studies, residence time VOIs were placed in the corresponding organs. The OLINDA/EXM program was used to estimate the absorbed radiation dose.

**Results:**

The radiopharmaceutical was well tolerated and no drug-related adverse effects were observed. No metabolites could be detected in blood (30 and 60 min p.i.) and urine (60 min p.i.). [^68^Ga]NODAGA-RGD showed rapid and predominantly renal elimination. Background radioactivity in blood, intestine, lung, and muscle tissue was low (%ID/l 60 min p.i. was 0.56 ± 0.43, 0.54 ± 0.39, 0.22 ± 0.05, and 0.16 ± 0.8, respectively). The calculated effective dose was 21.5 ± 5.4 μSv/MBq, and the highest absorbed radiation dose was found for the urinary bladder wall (0.26 ± 0.09 mSv/MBq). No increased uptake of the tracer was found in HCC compared with the background liver tissue.

**Conclusions:**

[^68^Ga]NODAGA-RGD uptake in the HCCs lesions was not sufficient to use this tracer for imaging these tumors. [^68^Ga]NODAGA-RGD was well tolerated and metabolically stable. Due to rapid renal excretion, background radioactivity was low in most of the body, resulting in low radiation burden and indicating the potential of [^68^Ga]NODAGA-RGD PET for non-invasive determination of integrin α_v_β_3_ expression.

## Introduction

Hepatocellular carcinoma (HCC) is one of the most common cancers worldwide, and liver cirrhosis is its primary risk factor [1]. The diagnosis of HCC is based on pathology or, in cirrhotic patients, on typical hallmarks in dynamic contrast-enhanced computed tomography (CT) or magnetic resonance imaging (MRI) with hypervascularization in the arterial and wash-out in the portal venous phase [2]. Early diagnosis is important, as curative treatment options, including resection, loco-ablative procedures, and liver transplantation are reserved for patients with early tumor stages without extrahepatic spread. Also assessment of treatment response and early detection of recurrent disease after (loco-)ablative therapy rely on non-invasive CT and MRI criteria [3].

However, definite diagnosis is not always possible with the currently available non-invasive methods. Especially in small lesions <1 cm contrast enhancement can be atypical and difficult to assess. Furthermore, a clear distinction between vital and devitalized tissue after loco-ablative treatment is not always possible. Therefore, additional functional imaging techniques for diagnosis and evaluation of treatment response in HCC are needed.

The use of positron emission tomography (PET) as an alternative non-invasive method has not been implemented in routine HCC diagnostics due to a lack of HCC-specific tracers. Recently, non-invasive imaging of integrin α_v_β_3_ expression using PET was introduced. This integrin is highly expressed on activated endothelial cells during angiogenesis and is involved in tumor growth and invasiveness [4]. Modified RGD peptides (RGD = amino acid sequence arginine–glycine–aspartic acid) are used as radioactive tracers binding to this integrin [5, 6]. One of the most extensively studied derivatives is [^18^F]Galacto-RGD [7–10]. Studies in animal models and clinical studies have demonstrated receptor-specific accumulation, as well as high metabolic stability and predominantly renal elimination [7, 9]. The routine use of this compound in clinical practice is hampered by its complex synthesis, preventing an automated production. An alternative, more easily accessible derivative is [^68^Ga]NODAGA-RGD [6, 11, 12], which shows a comparable receptor-specific accumulation and pharmacodynamics in preclinical studies, but has the advantage of an easy and automatable production.

[^68^Ga]NODAGA-RGD has a high binding affinity for integrin α_v_β_3_, which is upregulated on cytokine-activated endothelia cells and on vascular cells within malignant tumors [4]. Recent findings from immunohistochemical studies report integrin α_v_β_3_ expression in 77 % of investigated HCC specimens whereas expression was detectable only in 22 % of normal liver tissue [13]. Patients with detectable integrin α_v_β_3_ expression had a significantly worse survival, indicating a prognostic role of this integrin in patients with HCC.

For evaluation of the safety and diagnostic utility of [^68^Ga]NODAGA-RGD PET for HCC, this phase I clinical study was carried out. To characterize the biological properties of this novel tracer, whole-body biodistribution, pharmacokinetics, and metabolic stability of [^68^Ga]NODAGA-RGD in humans were determined. From these parameters, radiation dosimetry of a [^68^Ga]NODAGA-RGD PET investigation was calculated.

## Materials and methods

If not otherwise indicated, reagents were obtained from VWR International GmbH (Vienna, Austria) or Sigma-Aldrich Handels GmbH (Vienna, Austria) and were used without further purification.

### Tracer production via automated synthesis

^68^Ga-labeling of NODAGA-RGD follows the protocol published in Knetsch et al. [11] and was adapted to be carried out with a remote controlled synthesis unit (Modular-Lab PharmTracer; Eckert&Ziegler Eurotop GmbH, Berlin, Germany) with removable cassettes under cleanroom conditions. For the automated synthesis, a fractionated elution protocol was used [14]. Briefly, preparation of [^68^Ga]NODAGA-RGD starts with the fractionated elution of the ^68^Ga/^68^Ge-generator with 0.1 N HCl followed by reaction of ^68^GaCl_3_ with 20 μg NODAGA-RGD (MW: 960.5 g/mol) in 1.5 ml acetate buffer (2 M; pH 5.0) at 40 °C for approx. 10 min and subsequent adsorption of the product on a C-18 cartridge. Elution with 50 % ethanol and washing with saline including sterile filtration using a Millex GS (Millipore GmbH, Vienna, Austria) sterile filter with 0.22-μm pore size resulted in the desired radiolabeled product in approx. 8.5 ml 0.9 % saline with max. 10 % ethanol. NODAGA-RGD was supplied from piCHEM (Graz, Austria) in GMP quality. The ^68^Ge/^68^Ga-generator was purchased from Eckert&Ziegler Eurotop GmbH and eluted with 0.1 N hydrochloric acid (Rotem Industries Ltd, Arava, Israel).

### Patients

The study included nine patients, and was approved by the ethics committee of the Medical University of Innsbruck and the Austrian Competent Authority (BASG, EudraCT No. 2013-003741-42). Informed written consent was obtained from all patients. Inclusion criteria were untreated HCC lesions in patients with liver cirrhosis Child–Pugh class A or B. Diagnosis and exact number and size of HCC lesions was confirmed by a multiphasic CT or MRI according to EASL/EORTC guidelines [2]. Exclusion criteria were decompensated liver cirrhosis Child–Pugh class C, uncontrolled complications of portal hypertension (refractory ascites, advanced hepatic encephalopathy or large esophageal varices), and advanced renal insufficiency with an eGFR below 30 ml/min. Baseline examinations included CT or MRI scan, physical examination, ECG, and laboratory tests (including creatinine, blood count, transaminases, bilirubin, and coagulation parameters) not older than 14 days prior to the PET scan. Physical examination and laboratory tests were repeated on the day after the PET scan and during further follow-up visits to assess possible adverse reactions.

### PET procedure

For each patient, a total of three PET/CT scans were performed using a Discovery PET/CT 690 VCT scanner (GE Healthcare, Milwaukee, WI, USA). The patients were allowed to urinate after the second scan and a sample of the urine was collected for metabolic analysis. For all patients, the region from upper thigh to the skull/cranium was covered by a seven-bed emission scan (2 min per bed position; field of view is 15.2 cm with overlapping acquisitions, resulting in a length of 12.3 cm for each bed position) performed in caudocranial direction. The mean starting times of the three scans were 8.3 ± 2.1, 36.9 ± 2.8, and 75.1 ± 3.4 min after tracer injection, respectively. Injected activity ranged from 154 to 184 MBq [^68^Ga]NODAGA-RGD corresponding with approx. 10–12 μg peptide. X-ray CT transmission scans were performed twice, before the first and the third scan, to correct for gamma ray attenuation and to obtain the anatomical data required for drawing of the volume of interest (VOI). A second CT scan was required to guarantee the same positioning of VOIs (as for first and second scan) for image analysis because of patient movement.

### Image analysis and dosimetry

Positron emission data were reconstructed using an ordered-subsets expectation maximization algorithm. Reconstructions were performed with two iterations and 24 subsets. The images were corrected for attenuation using CT data collected over the same regions as for emission imaging. For image analysis, Hermes software (Version P5 gold 4.4-B; Hermes Medical Solutions AB, Stockholm, Sweden) was used. Images were calibrated to Bq/ml for radiation dosimetry estimates.

To obtain the time activity curves and to calculate the residence times, VOIs were placed in the corresponding organs (liver, urine bladder, spleen, kidneys, small intestine, muscle, lung, and left ventricle). The diameter of the VOIs was set to 2.5 cm for all organs with the exception of the liver, where it was set to 5 cm and placed in the center of the corresponding organ to avoid underestimated organ radioactivity because of partial-volume effects. Due to patient movement, the PET/CTs were fused between second and third scan to guarantee the identical position of VOIs at different scans.

Because of the limited number of measured time points, a monoexponential fit based on the decay corrected data was used for calculation of organ residence times.

For dosimetry calculations, the OLINDA/EXM software (Version 1.1, copyright Vanderbilt University, 2007) was used. Radioactivity in the source organs was determined by multiplying the measured radioactivity concentration (Bq/ml) by the organ masses from the OLINDA adult men phantom; the data were body weight- and lean body mass index-corrected; furthermore, kidney mass was patient-corrected by CT scan.

Urinary bladder radioactivity was determined from three VOIs at least 2.5 cm in diameter drawn inside the bladder. Subsequently, the urinary bladder volume was measured by drawing freehand ROIs around bladder contour at each of the three emission scans. The volume was calculated by HERMES software. The results were used to calculate the urine bladder content residence time.

### Analysis of the metabolic stability in blood and urine

Blood samples were collected 30 and 60 min and urine 60 min after tracer injection. Blood samples (approx. 15 ml) were centrifuged at 2,500 rpm for 5 min. The supernatant was treated with acetonitrile (1:1) and centrifuged at 3,000 rpm for 3 min. To remove the organic solvent, 1 ml of the supernatant was treated with an argon stream and approx. 300 μl of the remaining solution was analyzed. For urine analysis, an aliquot of 1 ml was passed through a sterile filter (Millex GV, Merck KGaA, Darmstadt, Germany), washed with 1 ml water (which was separately collected) and 50 μl of the filtrate was analyzed.

Analysis of metabolites was carried out using reversed-phase high-performance liquid chromatography (RP-HPLC) systems. For blood sample analysis, a Dionex P680 Pump (Germering, Germany) with a sample loop of 1 ml and a SRD Nucleosil 120-3C18 column (150 × 3 mm; Vienna, Austria) were used and fractions of 30 s were collected manually and counted in a 2480 Wizard^2^ 3″ automatic gamma counter (PerkinElmer, Vienna, Austria). For urine analysis, a Dionex Ultimate 3000 RS with an ACE 3C18 column (150 × 3 mm; Aberdeen, Great Britain) and a raytest Gabi radiometric detector (raytest Isotopenmessgeraete GmbH, Straubenhardt, Germany) were used. For both HPLC systems, an acetonitrile/water/0.1 % trifluoroacetic acid gradient was used (0–2 min 0 % acetonitrile, 2–18 min 0–50 % acetonitrile; flow 1 ml/min).

For determination of the extraction efficiency, aliquots during the different working steps were taken, analyzed in the gamma counter, and radioactivity distribution was calculated.

## Results

### Tracer production

The automated synthesis allowed production of [^68^Ga]NODAGA-RGD including prearrangement and quality control within approx. 60 min. All quality control parameters were within the pre-specified limits. This included half-life, appearance, pH value, identity, sterility, endotoxin amount, ^68^Ge-content, and ethanol content (see Table 1). Moreover, radiochemical purity based on HPLC as well as thin-layer chromatography (TLC; methanol/ammonium acetate 1:1) analysis was always >99 %. The specific activity is determined by the radioactivity eluted from the generator and was between 12 and 24 MBq/nmol (average 16.2 ± 3.3 MBq/nmol).

**Table 1:** Quality control data from a representative synthesis run

Determined parameter	Defined range	Found value
Half life	61.2–74.8 min	66.2 min
Appearance	Clear colorless	ok
Volume	7–12 ml	8.5 ml
Particle	Free of particle	ok
pH	4.5–7.0	6
Radiochemical purity TLC	>98 %	99.7 %
Radiochemical purity HPLC	>92 %	99.4 %
Identity HPLC (retention time)	6.7–7.3 min	7.0 min
Peptide amount (HPLC)	<25 μg	14 μg
Sterility test^a^	Sterile	ok
^68^Ge content^a^	<100 Bq/ml	0.02 Bq/ml
Endotoxin	<14.6 EU/ml	<0.25EU/ml
Ethanol content (GC)^a^	<10 %	2.7 %

^a^Has been carried out after release of the radiopharmaceutical

**Table 2:** Patient data (ES = emission scan)

Patient no.	Age [years]	Weight [kg]	Underlying liver disease	MELD score	No. of HCC lesions	Max. diameter [mm]	Injected activity [MBq]	Time after injection [min]		
								ES 1^a^	ES 2	ES 3
1	61	73	Fatty liver	10	2	20	167	8	37	72
2	55	96	Fatty liver	7	6	18	154	8	34	78
3	56	72	Fatty liver	9	3	28	173	13	40	76
4 (-)^b^	56	91	Fatty liver	10	2	33	167	8	34	72
5	63	89	Hepatitis C	8	1	34	175	7	35	70
6	75	133	Fatty liver	8	1	15	155	8	35	80
7	70	65	Fatty liver	8	3	20	184	10	40	77
8	52	97	Hepatitis C	10	2	20	159	7	36	78
9 (-)^b^	52	80	Hepatitis C	7	3	31	167	6	41	73
mean	60	88					167	8	37	75
SD	8	20					10	2	3	4

^a^Start of corresponding emission scan

^b^Indicates patient with lower uptake in HCC than surrounding liver tissue

### Patients, tolerability, and adverse effects

Nine male patients (mean age, 60 years, range, 52–75 years; Table 2) underwent a [^68^Ga]NODAGA-RGD scan. The etiology of the underlying liver disease was fatty liver disease in six patients and chronic hepatitis C in three patients. All patients had compensated cirrhosis Child–Pugh class A with a median MELD score of 8 (range, 7–10). The median number of HCC lesions was 2 (range, 1–6) and the median size of the largest lesion was 20 mm (range, 15–34 mm).

Tracer application and PET scanning were well tolerated in all patients, and no procedure-related adverse reactions were observed. During follow-up, no significant changes in kidney, liver, or heart function were revealed by clinical and biochemical investigations. All patients were alive at the end of the study period.

### Metabolite analysis

**Fig. 1:**
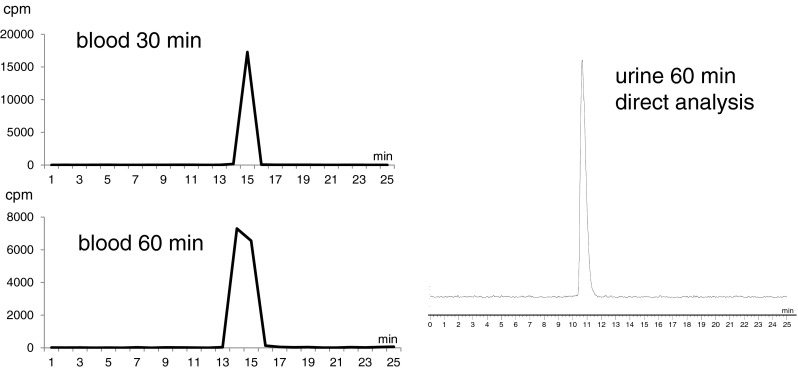
Metabolite analysis via HPLC. *Left* Analysis of the blood via fractionation and subsequent measurement of the fractions in a gamma counter. *Right* Analysis of the urine directly with the radiodetector of the HPLC system

No metabolites were detected by HPLC analysis of the blood samples drawn at 30 and 60 min (*n* = 3) and urine samples at 60 min (*n* = 6) after tracer application (Fig. 1). Extraction efficiency for the analysis of the blood samples was approx. 95 % and for the analysis of the urine samples greater than 99.5 %.

Due to the low radioactivity concentration in blood, HPLC radiodetectors were not able to analyze the samples, thus 30-s fractions were collected and radioactivity in the fractions was analyzed using a gamma counter.

### Pharmacokinetics and tumor accumulation

**Fig. 2:**
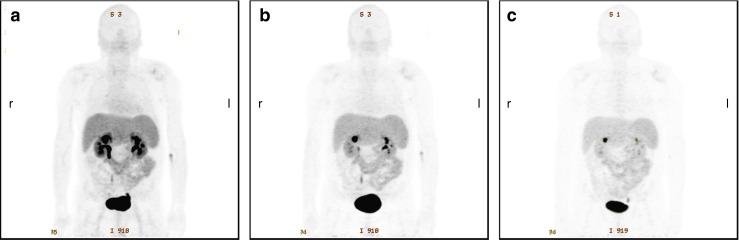
Maximum intensity projections from static [^68^Ga]NODAGA-RGD PET scans of a male patient (no. 3) with HCC starting at 13 min (**a**), 40 min (**b**), and 76 min (**c**) after tracer injection. The tracer shows rapid predominant renal elimination with highest radioactivity in bladder, kidneys, liver, spleen, and intestine. Low background radioactivity is found in brain, thorax, and extremities. For all three images, gray scale is set to the same values

**Fig. 3:**
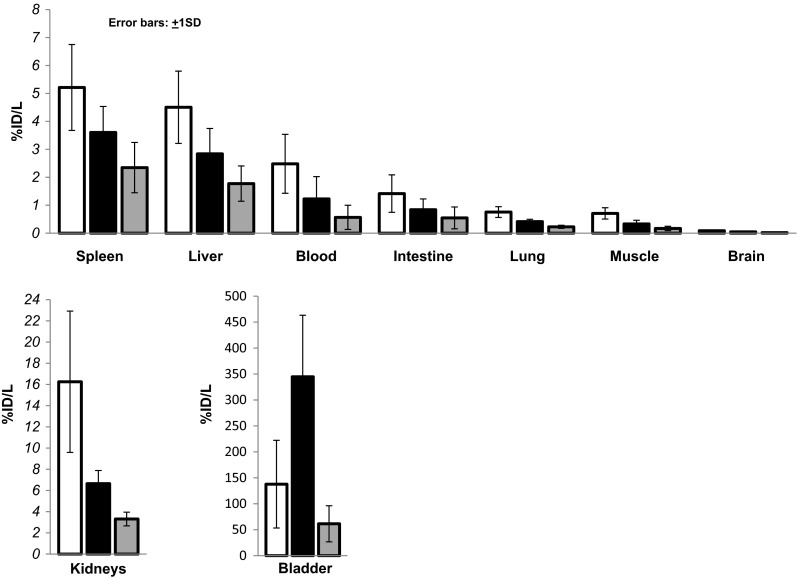
Biodistribution data of [^68^Ga]NODAGA-RGD from major organs and tissue. Mean percentage injected radioactivity per liter is given (%ID/l). Data are extracted from the three static PET scans corrected for decay. *White bar* = 8 ± 2 min p.i.; *black bar* = 37 ± 3 min p.i.; *gray bar* = 75 ± 4 min p.i. Due to the great differences of the determined values, three different *y*-axes are included

**Fig. 4:**
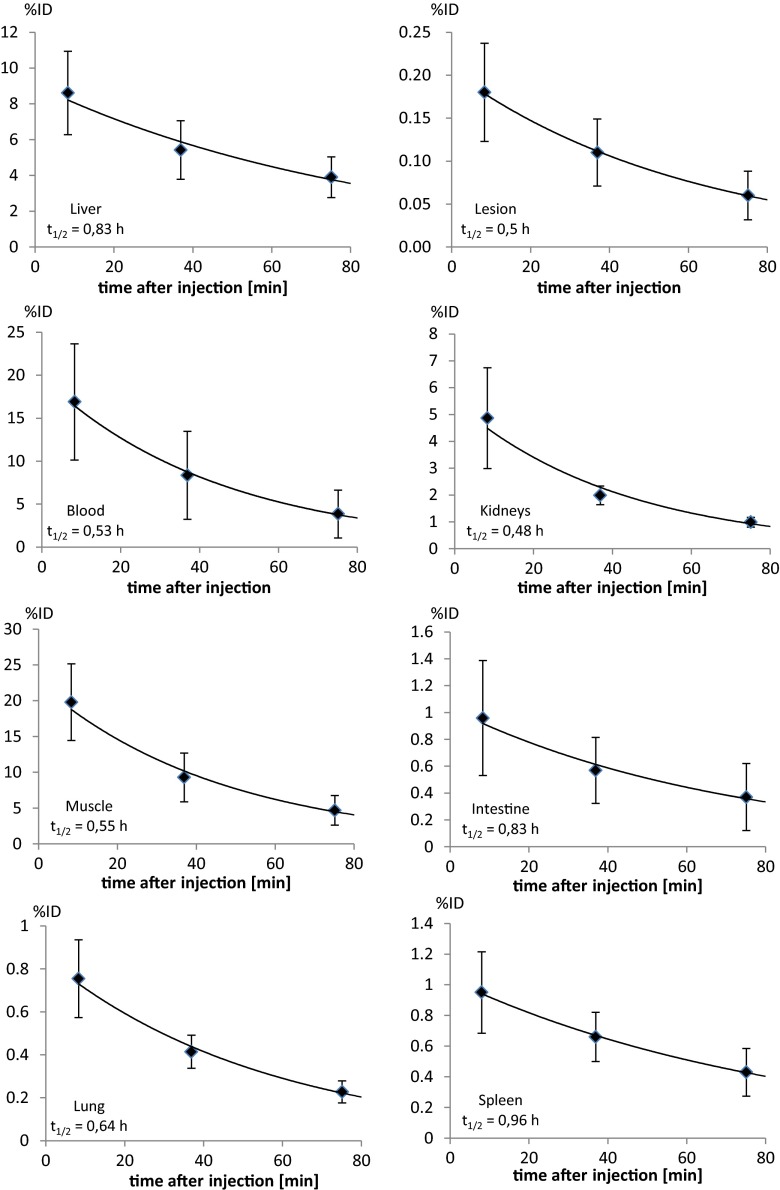
Organ time–activity curves and corresponding effective half-lives for [^68^Ga]NODAGA-RGD averaged over all patients. The graphs present the percentage of total injected radioactivity in each organ/tissue vs. time after tracer injection. Additionally, the averaged TAC of the lesions with deficit uptake including two patients was presented (*top right*). Uptake/elimination of the other lesions (altogether seven patients) followed the TAC of the liver (*top left*)

Figure 2 shows representative maximum intensity projections of three static scans from patient no. 3 starting 13, 40, and 76 min p.i., respectively. The images indicate rapid and predominantly renal elimination of [^68^Ga]NODAGA-RGD with highest radioactivity concentration in bladder, kidneys, spleen, and liver (median SUV 60 min p.i. = 31.0, 4.5, 3.8, and 2.9, respectively). Radioactivity in other tissue and blood is low (e.g., median SUV 60 min p.i. in intestine, blood, lung and muscle is 0.88, 0.72, 0.39, and 0.26, respectively). This is confirmed by quantitative analysis of the organ distribution as well as the time activity curves. Figure 3 summarizes the percentage injected dose per liter (%ID/l) for the various organs determined and averaged over all patients. Additionally, averaged time activity curves including all nine patients were calculated (Fig. 4). Again, the areas of highest radioactivity were the urogenital tract (kidneys and urinary bladder), followed by the spleen, liver, and gut. Blood-pool radioactivity was low and declined rapidly over time. Background radioactivity in the muscles and lungs was also low.

**Fig. 5:**
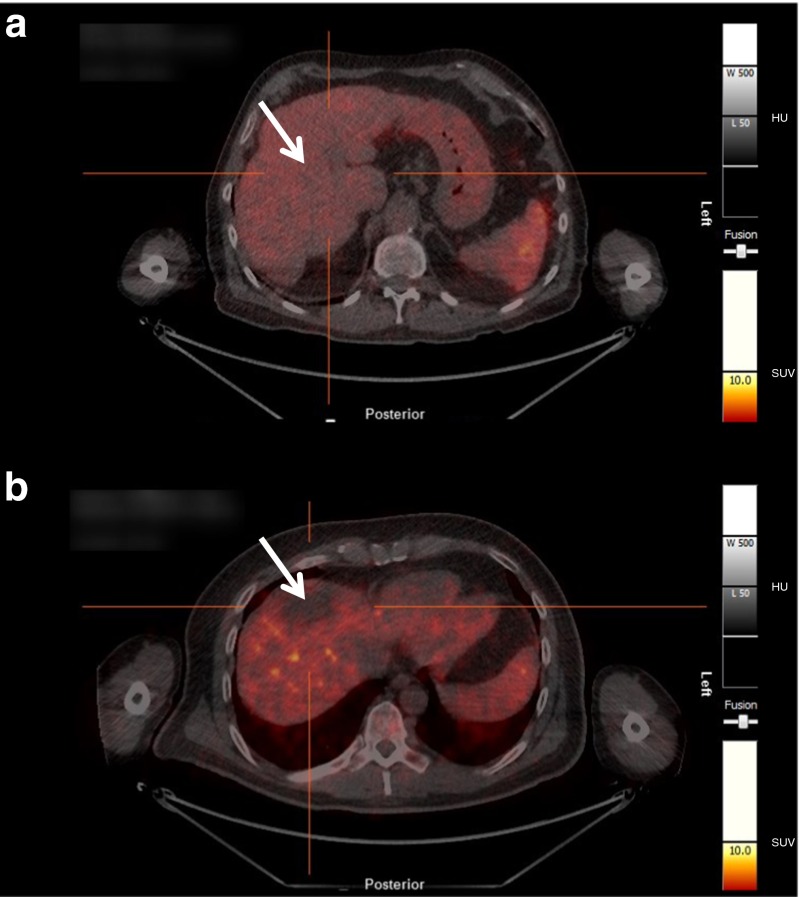
Transaxial PET/CT images of the tumor region. **a** Patient 7: Tracer uptake in the tumor is comparable with background radioactivity of the liver. **b** Patient 4: A deficit uptake is found in the lesion compared to the background radioactivity of the liver. *Arrows* indicate the position of the lesions

There was no increased radioactivity concentration in any of the HCC lesions identified by CT/MRI scan compared to the background radioactivity in the liver. In contrast, two out of nine patients showed a lower tracer accumulation in the HCC lesion as compared to the remaining liver parenchyma. Figure 5 shows representative images of two patients, one with comparable tracer accumulation in tumor and liver (panel A) and one with decreased tracer accumulation in the tumor compared to the rest of the liver (panel B).

### Dosimetry calculations

Table 3 lists the average absorbed radiation dose for all organs using individual residence times. Average residence times are listed in Table 4. The maximum residence time for ^68^Ga is 1.64 h, and the sum of the mean residence time is 1.11 h. The difference accounts for radioactivity excreted via the kidneys and urinary bladder by voiding after the second scan. The effective absorbed radiation dose was 21.5 ± 5.4 μSv/MBq with the highest absorbed dose (0.26 ± 0.09 mSv/MBq) found in the urinary bladder wall. Further organs with values of >0.10 mSv/MBq were kidneys, spleen, liver, and small intestine. Using a 30-min bladder voiding model the effective dose was reduced to 10.9 ± 1.9 μSv/MBq.

**Table 3:** Radiation dose estimates for intravenous administration of [^68^Ga]NODAGA-RGD in the order of increasing radiation burden. Data are given as μSv/MBq

Site	Mean	Standard deviation
Stomach wall	1.95	1.94
Breast	5.01	1.63
Skin	5.03	1.57
Brain	5.21	1.64
Thyroid	5.39	1.75
Red marrow	5.47	1.66
Thymus	5.57	1.77
Muscle	6.30	1.73
Heart wall	6.33	1.92
Testes	7.15	1.77
Lungs	7.17	1.71
Upper large intestinal wall	7.75	2.01
Adrenals	7.88	2.05
Pancreas	7.99	2.14
Osteogenic cells	8.10	3.05
Gallbladder wall	8.67	2.29
Lower large intestinal wall	8.98	2.09
Small intestinal wall	15.80	5.56
Liver	32.00	10.75
Spleen	48.20	17.12
Kidneys	69.00	17.37
Urinary bladder wall	262.00	92.59
Total body	7.29	2.20
Effective dose	21.50	5.35

**Table 4:** Mean organ/tissue residence times of all patients given as (MBq × h/MBq) × 10^2^

Site	Mean	Standard deviation
Muscle	24.6	8.1
Blood	1.1	0.5
Lungs	1.1	0.2
Small intestine	1.6	0.9
Liver	11.3	3.6
Spleen	1.8	0.6
Kidneys	5.4	1.0
Urinary bladder wall	22.3	8.0
Remainder of body	42.2	13.1

## Discussion

This phase I clinical study demonstrates that [^68^Ga]NODAGA-RGD is well tolerated without drug-related adverse effects in patients with liver cirrhosis and HCC. The good tolerability of this compound is in accordance with previously reports of RGD-based tracers [15, 16].

[^68^Ga]NODAGA-RGD showed high metabolic stability. No metabolites could be detected in blood or in urine samples. This indicates that the radioactivity accumulation solely correlates with [^68^Ga]NODAGA-RGD distribution. Our findings suggest superior stability compared to some other RGD-based tracer such as e.g., [^18^F]Fluciclatide, which showed only 74 % intact tracer in blood 60 min after injection [15]. Additionally, [^68^Ga]NODAGA-RGD showed rapid, predominantly renal elimination with low radioactivity concentration in almost all organs, resulting in an effective radiation dose similar to a standard [^18^F]FDG PET scan. Therefore, [^68^Ga]NODAGA-RGD can be used safely for non-invasive determination of integrin α_v_β_3_ expression in humans.

The dosimetry calculations showed an estimated effective dose of 21.5 μSv/MBq. The urinary bladder received the highest absorbed radiation dose (262 μSv/MBq). Other organs receiving more than 10 μSv/MBq were the small intestine, liver, spleen, and kidneys. In accordance with the data for [^18^F]Galacto-RGD, no time-dependent increase in radiation dose was present in the intestine. This indicates that the tracer uptake in the intestine is not due to hepatobiliary excretion, but rather due to receptor expression on intestinal smooth muscle cells as proposed by Beer et al. [10].

The radiation dose estimates from this study indicate low radiation burden for patients during a [^68^Ga]NODAGA-RGD PET scan. The total effective dose was in the range of other radiolabeled RGD-peptides used in clinical studies (e.g., [^18^F]Galacto-RGD, Fluciclatide, and RGD-K5; for overview see [6]) as well as other commonly used oncologic PET tracers such as [^18^F]FDG (between 20 and 30 μSv/MBq [17]). Even if 200 MBq [^68^Ga]NODAGA-RGD (average in this study was 167 ± 10 MBq) would have been injected, the effective dose would have only been 4.3 mSv. This is well within the limits of risk category IIB as defined by the International Commission on Radiological Protection (minor to intermediate level of risk, appropriate for intermediate to moderate social benefit) [18]. To further reduce exposure to the bladder, patients were allowed to void between the second and third static scan in this study (corresponding to a voiding interval of 60 min p.i.). This reduces radioactivity concentration in the bladder to approx. 20 % of the value found before voiding (see Fig. 3). The time–activity curves from other organs indicate that their radioactivity elimination is unimpaired, because no unpredicted curve progression between the second and third time point was observed. The radiation burden could be further reduced by using only one static scan after approximately 1 h in a routine application of [^68^Ga]NODAGA-RGD, which allows voiding already 30 min after tracer injection. Based on a corresponding voiding model, this would result in an effective dose which is approx. 50 % of that found in this study.

Analysis of biodistribution revealed predominantly renal elimination with highest radioactivity concentration in bladder followed by the kidneys. Due to the high radioactivity concentration in the urine, image analysis adjacent to urinary tract and the bladder might be slightly impaired especially in early scans. However, in the latest scan, due to rapid elimination and voiding, this is much less pronounced, indicating that in the clinical routine, scans should start approx. 60 min after tracer injection.

In contrast, background radioactivity in muscle, lung, blood, and brain is very low, indicating high sensitivity of [^68^Ga]NODAGA-RGD in integrin α_v_β_3_ detection in brain, thorax, and extremities. In contrast, higher background radioactivity was found in the spleen and liver and to a lesser extent in the intestine. However, compared with the data for [^18^F]Galacto-RGD [10], these values are even lower, potentially enabling better imaging contrasts for [^68^Ga]NODAGA-RGD. Moreover, the mean SUV in the liver (average of all patients) was between 3.8 (first image) and 2.9 (last image), which is comparable with liver background radioactivity of a routine [^18^F]FDG scan.

Radioactivity accumulation was not increased in HCC lesions identified by CT or MRI scan as compared to the background liver uptake. In two patients, even reduced uptake in the lesions was found. This finding is surprising, as integrin α_v_β_3_ expression has been shown to be present in the majority of HCCs [13]. Similar to our finding, a deficit uptake of [^18^F]Fluciclatide in liver metastases in breast cancer patients has been reported [15]. In accordance with this finding, the angiogenic potential of breast adenocarcinoma liver metastases is low. In contrast, HCC is a highly vascularized tumor and integrin α_v_β_3_ expression is upregulated in vascular cells in tumors [4]. Therefore, we expected high [^68^Ga]NODAGA-RGD uptake in HCC. One potential limitation is that no biopsy samples for histochemical analysis were available from HCC patients included in this study. Hence, we cannot confirm the actual integrin α_v_β_3_ expression of the HCCs investigated in this series.

In conclusion, imaging of HCC tumors might not be possible with [^68^Ga]NODAGA-RGD. If this is based on low receptor expression and/or to higher background radioactivity in the cirrhotic liver remains unclear. Future studies in tumor entities with known high integrin α_v_β_3_ expression either on the tumor cells or the endothelial cells of the tumor vasculature are under way to further explore the diagnostic utility of [^68^Ga]NODAGA-RGD for non-invasive imaging of integrin α_v_β_3_ expression.

## Conclusions

This study demonstrates that [^68^Ga]NODAGA-RGD is well tolerated and metabolically stable in humans. Due to rapid, predominantly renal, elimination of the tracer background, activity is low in most of the body, which results in low radiation burden and should lead to good tumor/background ratios. Unfortunately, uptake in HCC tumors was not sufficient to use the tracer for imaging of this tumor type. Further studies will evaluate the potential of this compound in imaging integrin α_v_β_3_ expression using PET in other tumor entities.
